# Effect of the COVID-19 Pandemic and Isolation Measures on the Mental Health Well-Being of Sixth Form Grammar School Students in Cumbria

**DOI:** 10.1192/bjo.2025.10243

**Published:** 2025-06-20

**Authors:** Medhini Varma, Pulkit Kaushal

**Affiliations:** 1 Newcastle University Medical Student, Newcastle, United Kingdom; 2 Cumbria Northumberland Tyne and Wear Valley Trust, Carlisle, United Kingdom

## Abstract

**Aims:** COVID-19 affected many countries globally, including the UK, to which the UK responded by placing lockdown measures throughout the country. This meant that many people were restricted in their everyday lives, including students. This study is used to understand the impact of these measures on sixth form students.

**Methods:** The Warwick–Edinburgh Mental Wellbeing Scale was sent to students in a sixth-form grammar school in September 2021 to assess students’ mental well-being. A semi-structured proforma was then also sent in May 2022 to compare students’ experiences and mental health before and after the lockdown measures.

**Results:** On the well-being scale, 47.1% of sixth-form students scored below 44 (average to low mental well-being), while 31% of the 1st year sixth-form students and 27% of the 2nd year sixth-form students scored 40 and below (lower than average mental well-being). In the semi-structured proforma, 73.90% of students (n=69) experienced quarantine since the pandemic started. 69.10% of them felt that the quarantines negatively impacted their mental health. In response to the question ‘In your own words, what was the most difficult thing that you experienced during the COVID pandemic?’ (n=55), 5 general themes were identified: *Isolation, not socialising, loneliness, loss of teenage life/youth, and online learning.*

**Conclusion:** The study showed that the mental well-being of sixth-form grammar school students in Cumbria was mostly negatively affected by the lockdown measures. The authors concluded that more accessible and approachable support should be provided to students in case of a similar event. More research is needed to understand the long-term impacts of such measures on students’ school and social lives.

